# Effectiveness of legislative changes obligating notification of prolonged sickness absence and assessment of remaining work ability on return to work and work participation: a natural experiment in Finland

**DOI:** 10.1136/oemed-2015-103131

**Published:** 2015-10-13

**Authors:** J I Halonen, S Solovieva, J Pentti, M Kivimäki, J Vahtera, E Viikari-Juntura

**Affiliations:** 1 Finnish Institute of Occupational Health, Helsinki, Finland; 2 Department of Epidemiology and Public Health, University College London Medical School, London, UK; 3 Faculty of Medicine, Clinicum, University of Helsinki, Helsinki, Finland; 4 Department of Public Health, University of Turku, Turku, Finland; 5 Turku University Hospital, Turku, Finland

**Keywords:** Return to work

## Abstract

**Objectives:**

Policies have been introduced to reduce sickness absence, but their effectiveness is largely unknown. In a natural experiment, we examined effects of legislative changes on return to work and work participation.

**Methods:**

The source population consisted of up to 72 164 Finnish public sector employees with a permanent job contract in 2008–2011 (before) and in 2013–2014 (after). We used employees with a continuous sickness absence of at least 30 calendar-days (n=5708–6393), 60 compensated days (n=1481–1655) and 90 compensated days (n=766–932). We examined sustainable return to work (a minimum of 28 consecutive working days) with survival analysis as well as monthly work participation after a sickness absence, and annual gain in work participation after the intervention, using trajectory analyses.

**Results:**

Sustainable return to work after 60 days of sickness absence occurred earlier after the legislative changes (p value 0.017), although the effect reduced towards the end of the follow-up. There were no differences in return to work after a 30 or 90 days of sickness absence. The largest annual gain, postintervention versus preintervention, in monthly work participation was observed among employees with 60 days of sickness absence and was 230.9 person-years/10 000 employees. The corresponding annual gains among those with 30 days and 90 days of sickness absence were 51.8 and 39.6, respectively.

**Conclusions:**

Our findings suggest that the legislative changes, obligating early notification of prolonged sickness absences as well as assessment of remaining work ability and possibilities to continue working, may enhance sustainable return to work in the short term. Other measures will be needed to enhance work participation, especially in the long term.

**What this paper adds**

- Prolonged sickness absences are known to be expensive to the society and may have harmful individual-level effects on health.
- Policies have been introduced to reduce sickness absence, but their effectiveness is largely unknown.
- We observed that legislative changes in Finland targeting more timely notification of prolonged sickness absences as well as improvement of the assessments of remaining work ability and the possibilities to continue working may increase sustainable return to work and work participation in the short term.
- Further measures will be needed to increase work participation, especially in the long term.

Sickness absences (SA) are expensive to the society due to costs of sickness benefits and decreased labour market competitiveness.^1^ At the individual level, high SA rates and prolonged absences have been associated with increased preterm exit from work,^2^ ^3^ and have been found to predict poor general health and mortality.^4–6^ In industrialised countries, the leading causes of prolonged absences are musculoskeletal diseases and mental disorders.^7^ ^8^ During the past decade, awareness has been raised of the increasing amount of prolonged SA in many European countries. In the UK, for example, Dame Carol Black suggested that physicians should move from the ‘sick note’ to the ‘fit note’ in the certification of SA, that is, possibilities to continue working should be assessed at an early stage of work disability.^9^ A recent study suggested that the fit note—introduced in 2010—may have reduced the occurrence of long-term (>12 weeks) absences.^7^ Comparable national actions, aiming to reduce SA, include the Dutch Labour Capacity Act^10^ in 2006, the Danish Return-to-work Programme^11^ in 2008 and the Norwegian Inclusive Working Life Programme in 2001, which has been revised and extended in 2004, 2010 and 2014.^12^ However, there is little knowledge of the effectiveness of these actions in reducing SA or improving return to work (RTW) at the population level.^7^ ^13^

In Finland, the employer is entitled to require a medical certificate from the employee to confirm the right to paid absence from work due to disability. It is typical that an employee can be absent for 3 days without a medical certificate,^14^ but the certificate is required for all absences beyond 7 days. For salaried employees, the employer will continue to pay full salary—depending on the length of employment—usually from 2 weeks up to 2 months. The Social Insurance Institution will pay sickness benefit after 10 weekdays of absence up to 300 weekdays, if necessary. As long as the employer continues to pay the salary to the employee, the sickness benefit is paid to the employer, based on a bill from the employer. If the absence continues beyond 60 compensated days (corresponding to 81 calendar-days), the Social Insurance Institution requires a detailed medical certificate in order to negotiate possibilities for rehabilitation.

As of 1 June 2012, three changes were introduced to the Health Insurance Act and to the Occupational Health Service Act in Finland. One amendment obligates the employers to inform the occupational health service (OHS) provider whenever an employee has been ill for 30 or more calendar-days (30-day rule). The medical certificate for short-term SA was also modified with an addition of a specific section to include suggestions for work modifications or rehabilitation that could enhance RTW, resembling the fit note in the UK, although the forms of work modification and rehabilitation are not accurately specified in the certificate. At the same time, in the second amendment the deadline for the employer to send a bill on daily allowance to the Social Insurance Institution was preponed from 4 to 2 months (60-day rule). The third amendment was that although any physician can assess work disability and issue-related certificates, the Social Insurance Institution will start to require an assessment by an occupational physician, if work disability persists for more than 90 compensated days (corresponding to 116 calendar-days, 90-day rule). About 60% of these assessments are delivered to the Social Insurance Institution by the 90th compensated day. This assessment will include assessment of work disability, as well as assessment of remaining work ability and identification of potential work modifications and rehabilitation possibilities to prevent unnecessary prolongation of absence from work. The OHS has a coordinating role in negotiations about work modifications with the employee and the employer. The changes described above have been collectively called the ‘30–60–90 day rule’. This rule emphasises early notification of both the OHS and the Social Insurance Institution of prolonged SA as well as the collaboration of the employee, the OHS and the employer in the assessment of possibilities to continue working. In Finland, the coverage of OHSs for salaried employees is high, up to 90%.

In this natural experiment, we assessed RTW for persons with 30 calendar-days, or 60 or 90 compensated SA days before and after the legislative changes. We also compared the patterns of work participation before and after, and estimated gains in work participation after the legislative changes.

## Methods

### Study design

To assess the effectiveness of the legislative changes, we carried out a natural experiment with three repeated samples with 12 months of follow-up, using SA registers of 11 Finnish towns (ie, employers) that attended the Finnish Public Sector (FPS) study.^15^ These registers do not include diagnoses for the absences. We looked at two time periods: the period preceding (2010/2011, preintervention) and the period following (2013/2014, postintervention) the legislative changes. Information from the year 2012 was not used, as it was treated as a washout period (see online supplementary figure S1). Since in the FPS study population the overall SA rate decreased over time (see online supplementary table S1), we also used 2008/2009 as a reference period that was compared with 2010/2011.

For the preintervention period, accrual for SAs started from 1 January 2010 and ended when the absence had continued for at least 30, 81 (corresponding to 60 compensated) or 116 (corresponding to 90 compensated) calendar-days, the last day of accrual for the given durations of SA being 31 December 2010 (see online supplementary figure S1). The follow-up started from the 31st calendar-day, or the 61st or 91st compensated day of SA and the participants were followed for work participation up to 12 months, the last possible day being 31 December 2011. Similarly, for the postintervention period, accrual for SAs started from 1 January 2013 and ended when the absence had continued for at least 30 calendar-days, or 60 or 90 compensated days, the last day of accrual for the given durations of SA being 31 December 2013. The follow-up started from the 31st calendar-day or the 61st or 91st compensated day of SA and the participants were followed for work participation up to 12 months, the last possible day being 31 December 2014. The SA accrual and follow-up periods were defined similarly for the reference period.

### Study population

**Figure 1:**
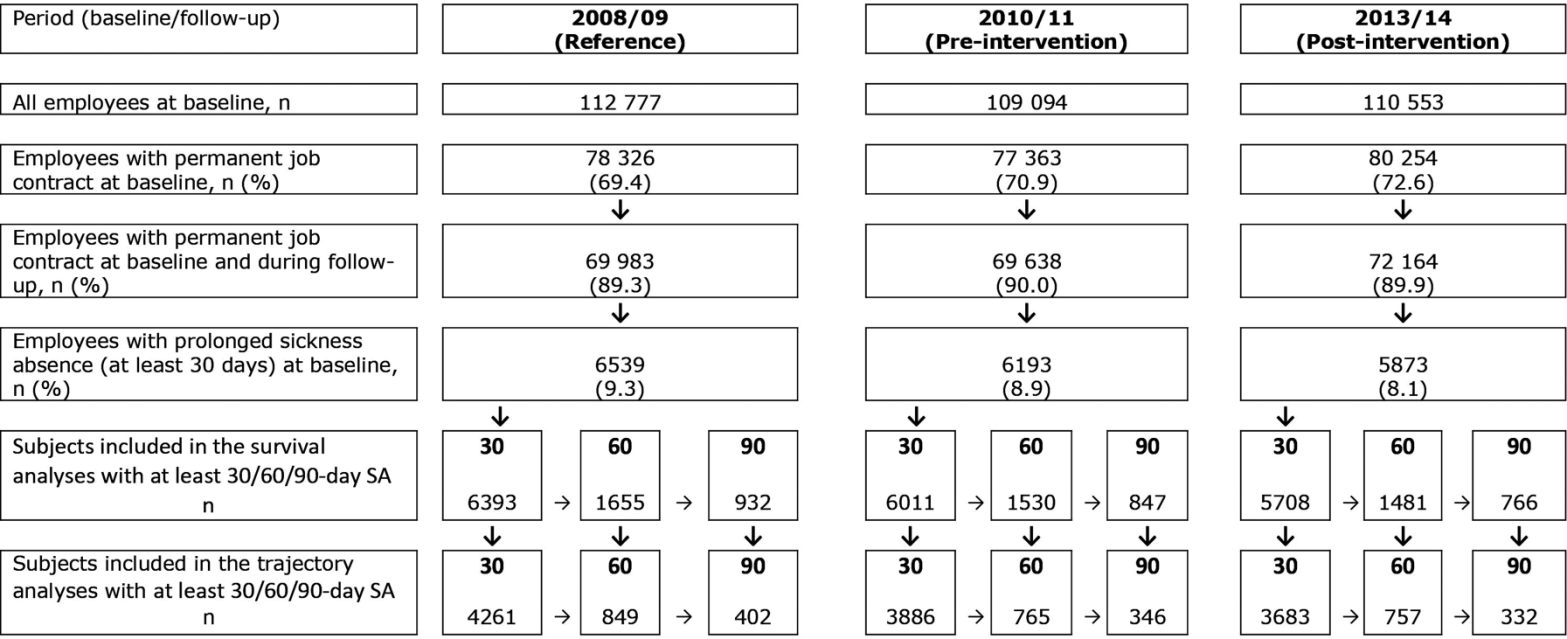
Flow chart of the sample formation for the three study periods and three lengths of sickness absence.

The source population included employees with a permanent job contract (approximately 70%) during the studied time periods in the 11 towns (figure 1). Most participants (approximately 75%) were women due to the nature of public sector jobs in Finland, nurses, practical nurses and teachers forming the largest occupational groups.

For the current analyses, we selected three populations with a continuous SA of at least 30 calendar-days, or 60 or 90 compensated days during the three accrual periods (2008, 2010 and 2013). We had information on the number of contracted days during each year. This number represented ‘days at risk’, which is the number of days an employee was supposed to be at work. From this measure, the number of days absent from work for reasons other than sickness (eg, holidays and maternity leave) was subtracted.^2^ We then excluded those who had more SA days than days at risk during the time period in question. To explore trajectories of monthly work participation after SA of 30, 60 or 90 days, we limited the sample to those whose employment contract covered the entire 12-month follow-up period.

### Outcomes

Complete information on all SA days was obtained from the employers’ registers for the years 2008–2014. Overlapping absence spells were excluded and subsequent absence spells were combined. For the analyses of RTW, the outcome was *sustainable RTW*, defined as a minimum of 28 consecutive working days following SA. To examine the trajectories of work participation, we used *monthly work participation* as an outcome, calculated as the proportion of days at work (not sick listed) during each 30 day period (ie, month) during the 12-month follow-up. A third outcome was *gain in annual work participation* from 2011 to 2014 that was measured in person-years/10 000 employees. As a reference, the gain from 2009 to 2011 was measured similarly.

### Covariates

Information on sex, age and occupational status (based on the International Standard Classification of Occupations, ISCO-08) was obtained from the employers’ registers. We dichotomised occupational status as: ‘high job status’ including managers, doctors, social workers, teachers (all have a university degree in Finland), nurses and office workers; and ‘low job status’ including occupations such as practical nurses, childminders and builders.

### Statistical analyses

#### Survival analysis

We used survival analysis (PROC LIFETEST in SAS) to plot RTW curves. Homogeneity of the survival curves was estimated using Wilcoxon (more sensitive when the ratio of hazards is higher at early survival times than at late ones) and log-rank (no weighing) tests. The follow-up of each subject started on the 31st calendar-day, or the 61st or 91st compensated SA day and ended with sustainable RTW, end of the job contract or end of follow-up, whichever came first. The follow-up time was counted in days.

#### Trajectory analysis

To examine the patterns of work participation during the 12-month follow-up after 30 calendar-days, or 60 or 90 compensated SA days, we used a semiparametric group-based modelling strategy by PROC TRAJ in SAS. This method was developed for identifying distinct groups of subjects who tend to have a similar profile over time (trajectories).^16^ The patterns of trajectories are defined by the analysis that uses posterior probability to assign the participants to the trajectory that best matches their behaviour (here monthly work participation) using all available data points from the follow-up period. We used the Bayesian information criterion (BIC) as the basis for selecting the number of trajectories.

#### Area under the curve analysis

To quantify the changes in work participation before and after the introduction of the legislative changes, we computed area under the curve (AUC) for each work participation trajectory using Trapezoidal Rule^17^ described by Shiang.^18^ We then calculated the differences in AUCs between the corresponding trajectories before and after the intervention (2011 vs 2014). After summing up the gain by trajectory pairs, we reported the total annual gain in work participation for the intervention. To assess the effect of the declining trend of SA, we additionally calculated annual gain in work participation between 2009 and 2011.

#### Effect modifiers and sensitivity analyses

To examine whether there are differences between specific population groups, we stratified the analyses of those with 60 compensated SA days by sex and by job status. We chose these groups a priori as higher absence rates are often reported for women than men,^19^ and for people with low versus high socioeconomic status.^20^ ^21^ As a sensitivity analysis, we examined separately a group of practical nurses, which is the largest group within the low job status category. With this analysis, we aimed to assess the potential effects of economic recession, as practical nurses are a group unlikely to have been affected by outsourcing activities due to the changes in economic situation during the intervention period. All analyses were performed with SAS software V.9.4.^22^

## Results

In the source population, the proportion of those with a short SA spell increased from 2008 to 2014, whereas the overall SA rate and the proportion of those with longer SA spells decreased (see online supplementary table S1). Descriptive statistics of the studied populations for the survival and trajectory analyses by the three time periods and length of SA are provided in table 1. Over the periods, there was a slight increase in the proportion of women, practical nurses as well as those with high job status.

**Table 1:** Descriptive statistics of the populations for survival and trajectory analyses by study periods and length of SA

Permanent workers	30-day SA* Study period			60-day SA† Study period			90-day SA† Study period		
Variable	2008/2009	2010/2011	2013/2014	2008/2009	2010/2011	2013/2014	2008/2009	2010/2011	2013/2014
Survival analyses (to plot sustained RTW‡ curves)	n=6393	n=6011	n=5708	n=1655	n=1530	n=1481	n=932	n=847	n=766
Age, years (95% CI)	49.6 (49.3 to 49.8)	49.8 (49.5 to 50.0)	49.8 (49.5 to 50.0)	51.7 (51.2 to 52.2)	51.7 (51.2 to 52.1)	51.1 (50.7 to 51.6)	52.6 (52.1 to 53.1)	51.8 (51.2 to 52.4)	51.3 (50.7 to 52.0)
Women, n (%)	4857 (76.0)	4614 (76.8)	4498 (78.7)	1219 (73.7)	1140 (74.5)	1158 (78.2)	695 (74.6)	633 (74.7)	597 (78.0)
Low job status, n (%)	3761 (58.8)	3390 (56.4)	3059 (53.6)	1068 (64.5)	921 (60.2)	847 (57.2)	609 (65.3)	538 (63.5)	444 (58.0)
Practical nurses, n (%)	841 (13.2)	879 (14.6)	932 (16.3)	203 (12.3)	220 (14.4)	261 (17.6)	117 (12.6)	120 (14.2)	140 (18.3)
Trajectory analyses (to plot work participation and calculate gains in work participation)	n=4261	n=3886	n=3683	n=849	n=765	n=757	n=402	n=346	n=332
Age, years (95% CI)	52.3 (52.0 to 52.5)	50.5 (50.3 to 50.8)	50.7 (50.5 to 51.0)	51.0 (50.5 to 51.6)	50.9 (50.3 to 51.6)	51.5 (50.9 to 52.1)	51.8 (51.0 to 52.5)	50.2 (49.4 to 51.1)	51.0 (50.1 to 51.9)
Women, n (%)	3120 (73.2)	2855 (73.5)	2790 (75.8)	594 (70.0)	544 (71.1)	567 (74.9)	286 (71.1)	254 (73.4)	244 (73.5)
Low job status, n (%)	2507 (58.8)	2180 (56.1)	1955 (53.1)	546 (64.3)	464 (60.7)	439 (58.0)	260 (64.7)	214 (61.8)	197 (59.3)
Practical nurses, n (%)	527 (12.4)	504 (13.0)	518 (14.1)	96 (11.3)	108 (14.1)	120 (15.8)	49 (12.2)	45 (13.0)	56 (16.9)

*Absence in calendar-days.

**†**Compensated SA days.

**‡**Sustained RTW defined as a minimum of 28 consecutive days at work after SA.

RTW, return to work; SA, sickness absence.

### Survival analysis

**Figure 2:**
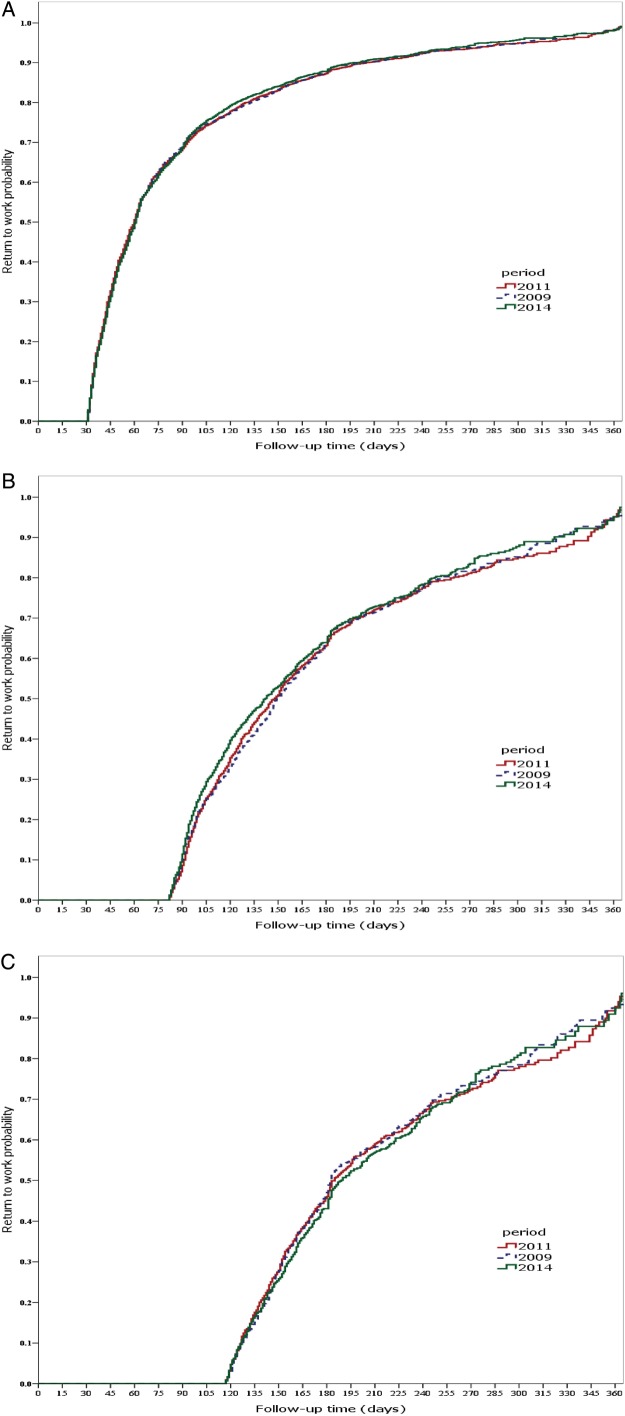
Plots for sustainable return to work after (A) 30 calendar, (B) 60 and (C) 90 compensated sickness absence days in the reference (2009), preintervention (2011) and postintervention (2014) periods.

According to survival analysis, sustainable RTW after 60 compensated SA days occurred earlier in 2014 than in 2011 (p value for the Wilcoxon test 0.017, figure 2B); however, the effect reduced towards the end of the follow-up (Sidak p value for the log-rank test 0.054). During the reference and preintervention periods, 50% of the participants had returned to work within 69 and 66 days, respectively, whereas the corresponding number of days for the postintervention period was 61 days. There were no statistically significant differences in RTW after a 30 calendar-day of SA, or after 90 compensated SA days (figure 2A, C, respectively). In the stratified analyses after 60 compensated SA days, women and those with low job status returned to work sooner in 2014 than in 2011 (Wilcoxon p value 0.0224/log-rank p value 0.1041 for women, and Wilcoxon p value 0.0062/log-rank p value 0.0399 for low job status). Among practical nurses, the difference in RTW between years was in line with the total sample though non-significant. No differences between 2014 and 2011 were observed in men or in those with a high job status (data not shown). Neither were there any differences in RTW between 2009 and 2011 in the total sample.

### Trajectory and AUC analyses

Participants included in the trajectory analyses (ie, those whose job contract continued over the 12-month follow-up) to examine patterns of work participation were less frequently women and they returned to work earlier than those who were excluded (whose contract did not cover the entire follow-up) (see online supplementary table S2). For those included with 60 compensated days, the median time for sustainable RTW was 50 days during the preintervention period and 41 days during the postintervention period.

**Figure 3:**
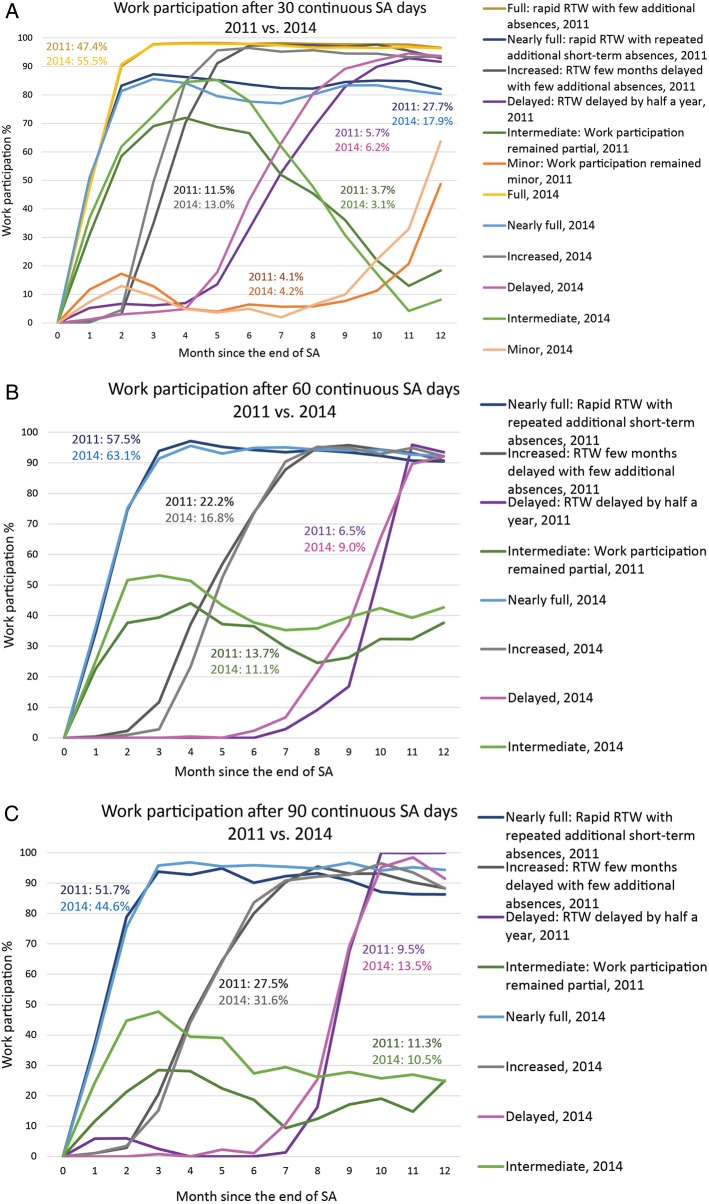
Trajectories for monthly work participation after (A) 30 calendar, (B) 60 and (C) 90 compensated SA days and annual gains in work participation between the preintervention and postintervention periods. Percentages represent the proportions of employees belonging to each trajectory (RTW, return to work, SA, sickness absence).

The trajectory analysis suggested the best fit for the six-trajectory model of work participation during the 12 months following a 30-day absence (figure 3A). ‘Full work participation’ includes those with a rapid RTW with only few additional absences after the 30-day SA; ‘nearly full work participation’, those with a rapid RTW but repeated short-term absences every month; ‘increased work participation’, those whose RTW was delayed a few months, after which only few additional absences occurred; ‘delayed work participation’, those whose RTW was delayed for about half a year; ‘intermediate work participation’, those whose work participation remained partial; and ‘minor work participation’, those whose work participation remained minor through the follow-up.

The largest trajectories by the proportion of employees both in 2011 (47.4%) and 2014 (55.5%) were those for ‘full work participation’. The second largest trajectories were those for ‘nearly full work participation’. The proportion of employees with this kind of pattern in work participation reduced substantially after the intervention. The total annual gain in work participation after the intervention in this group was 51.8 person-years/10 000 employees.

The trajectory analyses suggested the best fit for the four-trajectory models of work participation (‘full’, ‘increased’, ‘delayed’ and ‘intermediate’) during the 12 months following 60 or 90 compensated SA days (figure 3B, C). Among those with 60 compensated SA days, increase in work participation was seen in three of four trajectories, the gain being largest for the nearly full work participation, that is, those with a rapid RTW with some additional absences every month. The total annual gain in work participation after the intervention in those with 60 compensated SA days was 230.9 person-years/10 000 employees (ie, an approximately 2% gain). Similarly, among those with 90 compensated SA days, work participation increased in three of four trajectories. However, the largest gain was seen in the trajectories with RTW after several months (‘delayed work participation’). Among those with 90 compensated days, the total annual gain in work participation after the intervention was 39.6 person-years/10 000 employees.

**Figure 4:**
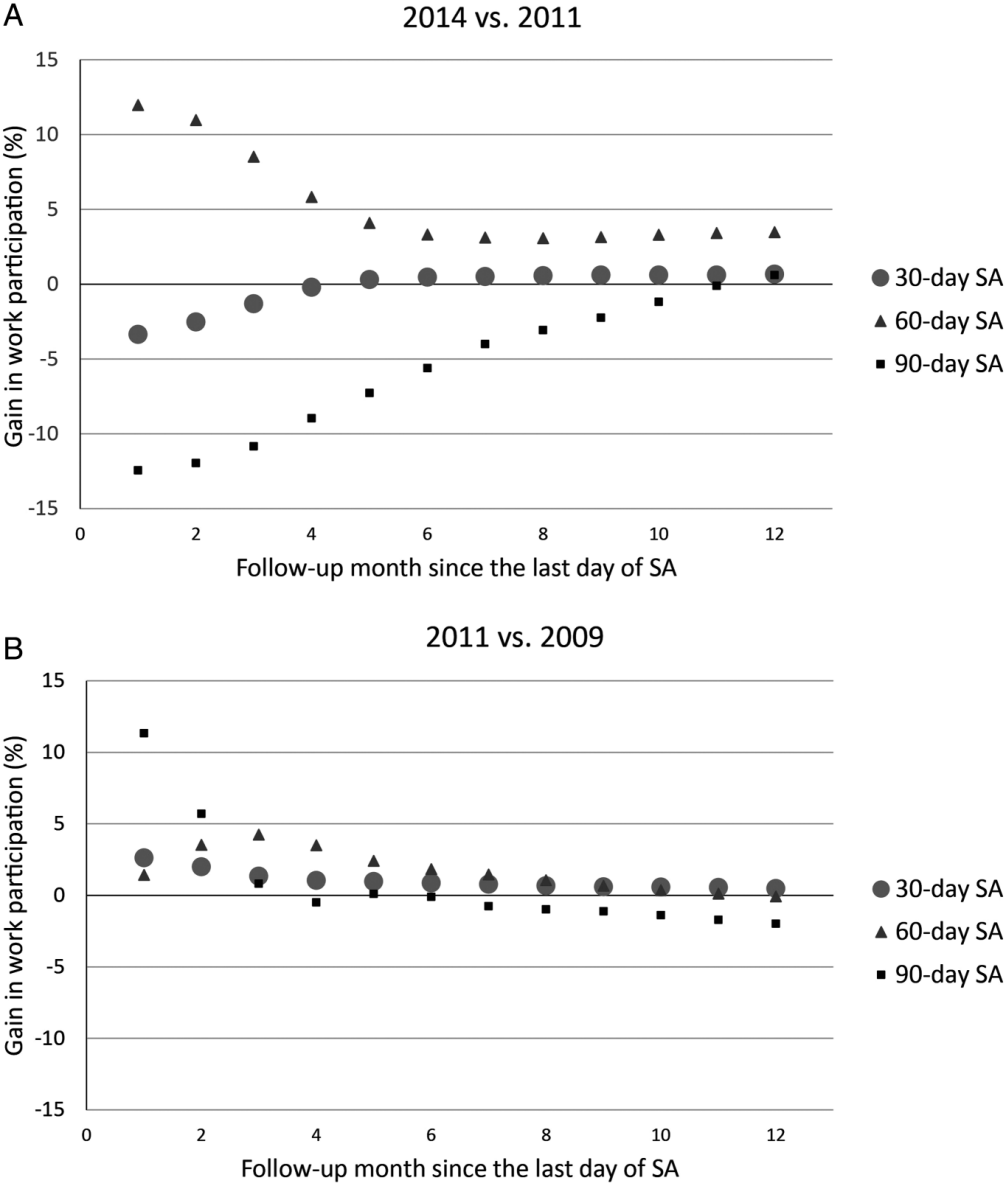
Monthly relative difference in work participation (A) between the postintervention and preintervention (2014 vs 2011) periods and (B) between the preintervention and reference (2011 vs 2009) periods by length of sickness absence. The size of the marker represents the size of the group being followed-up.

In figure 4A, we present monthly relative differences in work participation (%) between the follow-ups postintervention and preintervention for all three SA groups. An immediate intervention effect was seen for those with 60 compensated SA days. The effect declined during the first 5 months and stabilised towards the end of the follow-up. Among those with 30 calendar-days, work participation after the SA was smaller in the postintervention versus preintervention period until 5 months of follow-up. The same was seen among those with 90 compensated SA days until 10 months of follow-up. When the trajectory analyses were repeated for the time period 2008–2009, nearly similar patterns of work participation were seen in the three SA groups (data not shown). However, the annual gains from 2009 to 2011 were all smaller: 35.9, −4.9 and −128.7 person-years/10 000 employees after 30, 60 and 90 SA days, respectively. Relative differences in work participation in the reference period (between 2009 and 2011) were positive but small among those with an absence of 30 calendar days or 60 compensated days (figure 4B). The difference among those with a 90-day absence was more notable during the first 2 months, but it turned negative towards the end of the follow-up.

In the stratified trajectory analyses for those with 60 compensated SA days, in all but the high job status group the gains in work participation were positive for the intervention period. The largest annual gain in work participation was observed for the low job status group (409.7 person-years/10 000 employees), while for the high job status group the gain was negative (−30.4 person-years/10 000 employees). Women had a higher gain compared with men (287.8 vs 70.4 person-years/10 000 employees). In addition, the subgroup analyses for practical nurses showed an annual gain of 117.7 person-years/10 000 employees. Work participation was worse in 2011 for low job status, men and practical nurses compared to 2009 as the gains were negative (for 2011 vs 2009, the person-years/10 000 employees were for low job status −1091.5, high job status 137.6, women 35.5, men −81.3, and practical nurses −211.8).

## Discussion

These findings from the natural experiment suggest that changes in legislation targeting at more timely notification of prolonged SA may improve sustainable RTW. The changes included notification of the OHS and the national insurer, as well as the work capacity assessment with involvement of an occupational physician and the employer. The effects were most pronounced among those with 60 compensated absence days. However, time to sustainable RTW was slightly shorter after, than before, the legislative changes, and the overall postintervention gain in annual work participation was only about 2% in the group that had a work contract for at least 12 months after the absence. Altogether, our findings indicate that the legislative changes did enhance RTW, but this effect was diluted over time. Smaller net improvements after 90 than 60 compensated absence days, and the negative difference in relative change in work participation between preintervention and postintervention periods after 90 compensated days may imply that those able to continue working had returned to work earlier, and those with the most severe conditions continued their SA up to 90 days or more. We also observed larger gains in work participation among women than men, and among those with low compared with high job status.

The three legislative changes were implemented simultaneously, all aiming at the same outcome of enhancing RTW, and therefore it is not easy to distinguish the effects of the different components. First, the obligation of the employer to inform the OHS of SA exceeding 30 days, and of the national insurer of absences exceeding 2 months—by sending the bill on sickness benefits—both raise an awareness of prolonged absence. Second, the already existing requirement of a detailed medical certificate to the national insurer at the time of 60 compensated days as a basis for considering rehabilitation needs, with the new requirements to assess work capability before 90 compensated days in collaboration with the employer and the OHS, and to discuss possibilities for work modifications and part-time work, all point out to practical solutions to make RTW possible.

The public sector is characterised by rather stable work contracts. However, the total SA rates declined from 2008 until 2013 in both the public and the private sector. Owing to this decline before and during our intervention period, we compared the observed differences in work participation with those of the reference period. The gains in work participation days were larger during the intervention than the reference period, suggesting a beneficial effect of the legislative changes. The study was also carried out during an economic recession that is known to increase the risk of job termination and unemployment among employees with prolonged SA.^2^ This may also decrease SA through disciplinary effect, that is, risk of unemployment disciplines employees to be highly present.^23^ Therefore, we examined the group of practical nurses separately as their job market is less prone to general economic changes. We observed similar effects of the legislative changes among this subgroup compared with the total analysis sample, supporting that the findings are not fully explained by the recession.

### Comparison with other studies

There are few natural experiments that have examined the effectiveness of legislative changes on work participation at the population level.^8^ Our findings are in line with those suggesting beneficial effects of the introduction of the fit note on long-term SA in the UK.^7^ Moreover, a corresponding element with the Finnish occupational physician assessment, as part of the 90-day rule, has been suggested in the UK.^24^ In 2013, the UK Government agreed to fund an Independent Assessment Service, including a state-funded assessment by occupational health professionals for employees after 4 weeks of SA, suggestions of appropriate interventions, and providing employers and employees with advice on overcoming the barriers to RTW.^25^ ^26^ This may further enhance RTW in the UK.

The effects of our population-level intervention were modest. In a recent systematic review that assessed the effects of various measures targeted at enhancing RTW, there were no or mixed effects in the populations with non-specified SA.^27^ Given the heterogeneity of severity, prognosis and treatment options between health conditions and between possibilities for work modifications in a population with non-specified SAs, it is well conceivable that the responsiveness for a certain legislative change is limited. The optimal timing of structured interventions may also matter. Optimal timing has been estimated to be between 8 and 12 weeks for low back pain,^28^ but it may differ according to diagnosis. For a selected set of absence diagnoses, particularly musculoskeletal conditions, there is evidence supporting our findings that workplace interventions that use, for example, early contact with the worker by their workplace may reduce the duration of work disability.^28–30^ In our earlier population-level study on the effects of the introduction of the partial sick leave benefit in Finland, we found an overall 5% increase in work participation after 60 compensated SA days, while among those with mental disorders the increase was 2.5-fold.^8^

### Strengths and weaknesses of the study

This is among the first natural experiments assessing the effectiveness of legislative changes on work participation using a large study population from the public sector. However, some limitations need to be considered when interpreting these findings. One is that data for this study were collected shortly after the legislative changes became effective and their implementation may take time. Thus, all effects may not be seen immediately.

Limitations related to the sample selection include the exclusion of employees with a non-permanent job contract (approximately 30%), which prevented us from generalising our findings to the entire public sector as non-permanent employees (most with a short-term contract) may have less SA (≥1 week)^31^ and presenteeism^32^ than the long-term employees mostly with a permanent contract (ie, open-ended contract). We also dropped subjects with more absence than working days (approximately 3%). Since there were no differences in the background variables between the included and those with more absence than working days, this exclusion unlikely had any effects on the results. In the trajectory analyses, we included those subjects with a 12 month job contract following the prolonged SA. This group had higher percentages of sustainable RTW than the excluded group (roughly 10% difference), suggesting better responsiveness to the legislative changes compared to those whose job contract ended during the follow-up. We did not have information about the reasons for job termination; therefore, we were not able to examine whether the legislative changes affected the labour market participation in general. Since our sample was drawn from employers’ registers, we had no data on other factors that associate with SA and RTW such as medical condition. Finally, owing to the characteristics of the source data and the selection process, the generalisability of the findings to private sector employees, the self-employed and those with fixed-term contracts is limited.

The fact that we were not able to assess the effects of legislation on cause-specific SA prevented us from seeing diagnosis-specific differences. In some conditions, sustainable RTW may be enhanced with, for example, work modifications, whereas in severe conditions, sustainable RTW may not be possible under any work-related intervention. In such circumstances, no effect of intervention would be expected.

### Conclusions and policy implications

SA are expensive to the society and actions to improve sustainable RTW and work participation are needed. We observed in a large public sector population that changes in legislation that simultaneously obligated the employer to notify the OHS and the national insurer about prolonging SA as well as required assessments of work ability, and the possibilities to continue to work did enhance sustainable RTW in the short term. The effects were most pronounced after about 12 weeks (60 compensated days) of SA; however, the annual gain in work participation was modest. Therefore, other measures will be needed to increase work participation, especially in the long term. The effects of the studied legislative changes should be assessed in the private sector, and further research is needed to evaluate the monetary effects of these changes.

## Supplementary Material

Web supplement
