# Supplementary material for: Effectiveness of legislative changes obligating notification of prolonged sickness absence and assessment of remaining work ability on return to work and work participation: a natural experiment in Finland
Source: Occup Environ Med. 2015 Oct 13;73(1):42–50. doi: 10.1136/oemed-2015-103131 (PMC4717458; doi:10.1136/oemed-2015-103131)
Supplement: Web supplement [file oemed-2015-103131-s1.pdf]

Supplemental Material

Effectiveness of legislative changes obligating notification of prolonged sickness absence and assessment of remaining work ability on return to work and work participation: a natural experiment in Finland

J I Halonen, S Solovieva, J Pentti, M Kivimäki, J Vahtera, E Viikari-Juntura

Supplemental Table 1. Descriptive statistics of the source population with permanent job contract by years.

	**2008**	**2009**	**2010**	**2011**	**2012**	**2013**	**2014**
Total n	78 029	78 742	77 056	77 498	77 516	79 922	79 383
Age, mean years (95% CI)	46.8 (46.7-46.9)	46.9 (46.8-47.0)	47.1 (46.9-47.2)	47.0 (46.9-47.1)	46.6 (46.5-46.7)	47.0 (46.9-47.1)	47.2 (47.1-47.3)
Women, n (%)	57 741 (74.0)	58 742 (74.6)	58 023 (75.3)	58 511 (75.5)	58 982 (76.1)	60 821 (76.1)	60 648 (76.4)
Low job status, n (%)	46 694 (59.8)	47 403 (60.2)	46 390 (60.2)	47 028 (60.7)	49 300 (63.6)	50 136 (62.7)	49 884 (62.8)
Practical nurses, n (%)	7450 (9.8)	7846 (10.2)	7810 (10.3)	7916 (10.4)	8 185 (10.6)	8544 (11.0)	8530 (11.0)
Sickness absence rate, * % (95% CI)	5.4 (5.3-5.5)	5.0 (4.9-5.1)	5.1 (5.0-5.2)	5.1 (5.0-5.2)	4.8 (4.7-4.9)	4.7 (4.6-4.8)	4.5 (4.4-4.6)
Those, n (%) with							
At least one short absence (1-7 days)	49 002 (62.8)	50 474 (64.1)	49 470 (64.2)	49 289 (63.6)	50 075 (64.6)	50 910 (63.7)	51 440 (64.8)
At least one absence with 30+ days†	6790 (8.7)	6486 (8.2)	6502 (8.4)	6319 (8.2)	5 814 (7.5)	6065 (7.6)	5592 (7.0)
At least one absence with 60+ days‡	1925 (2.5)	1800 (2.3)	1844 (2.4)	1825 (2.4)	1 628 (2.1)	1776 (2.2)	1443 (1.8)
At least one absence with 90+ days‡	1203 (1.5)	1070 (1.4)	1096 (1.4)	1079 (1.4)	930 (1.2)	990 (1.2)	744 (0.9)

*Absence days/days at risk

†Calendar days

‡Compensated sickness absence days

Supplemental Table 2. Comparison of participants included in and excluded from trajectory analyses by study periods.

	Study period					
	2008/2009		2010/2011		2013/2014	
	Excluded	Included	Excluded	Included	Excluded	Included
30 calendar SA days						
Total n (%)	2132 (33.3)	4261 (66.7)	2125 (35.4)	3886 (64.6)	2025 (35.5)	3683 (64.5)
Age, mean years (95% CI)	48.2 (47.7-48.7)	50.3 (50.0-50.5)	48.3 (47.8-48.8)	50.5 (50.3-50.8)	48.0 (47.5-48.5)	50.7 (50.5-51.0)
Women, n (%)	1740 (81.5)	3120 (73.2)	1761 (82.8)	2885 (73.5)	1708 (84.2)	2790 (75.8)
Low job status, n (%)	1261 (59.1)	1923 (45.1)	1282 (60.3)	1822 (46.9)	1218 (60.1)	1849 (50.2)
Practical nurses, n (%)	314 (15.0)	527 (12.4)	375 (17.6)	504 (13.0)	414 (20.4)	518 (14.1)
Timing of sustainable RTW*, days (median, IQR†)	38.0 (13.0-118)	26.0 (10.0-61.0)	38.0 (12.0-118)	26.0 (10.0-62.0)	39.0 (14.0-110)	26.0 (10.0-63.0)
60 compensated SA days						
Total n (%)	806 (48.7)	849 (51.3)	765 (50.0)	765 (50.0)	724 (48.9)	757 (51.1)
Age, mean years (95% CI)	52.5 (51.8-53.1)	51.0 (50.5-51.6)	52.4 (51.7-53.1)	51.0 (50.3-51.6)	50.8 (50.0-51.5)	51.5 (50.9-52.1)
Women, n (%)	626 (77.6)	594 (70.0)	596 (77.9)	544 (71.1)	591 (81.6)	567 (74.9)
Low job status, n (%)	452 (56.1)	348 (42.0)	453 (59.2)	340 (44.4)	434 (59.9)	342 (45.1)
Practical nurses, n (%)	107 (13.3)	96 (11.3)	112 (14.6)	108 (14.1)	141 (19.5)	120 (15.8)
Timing of sustainable RTW*, days (median, IQR†)	93.0 (36.0-179)	54.0 (17.0-101)	85.0 (35.0-184)	50.0 (17.0-113)	78.0 (29.0-162)	41.0 (15.0-119)
90 compensated SA days						
Total n (%)	530 (56.9)	402 (43.1)	501 (59.1)	346 (40.9)	434 (56.7)	332 (43.3)
Age, mean years (95% CI)	53.2 (52.5-53.9)	51.8 (51.0-52.5)	52.8 (52.0-53.6)	50.2 (49.4-51.1)	51.6 (50.6-52.5)	51.0 (50.2-51.9)
Women, n (%)	410 (77.2)	286 (71.1)	379 (75.6)	254 (73.4)	353 (81.3)	244 (73.5)
Low job status, n (%)	290 (54.7)	162 (40.3)	279 (55.7)	142 (41.0)	264 (60.8)	144 (43.4)
Practical nurses, n (%)	68 (12.8)	49 (12.2)	75 (15.0)	45 (13.0)	84 (19.4)	56 (16.9)
Timing of sustainable RTW*, days (median, IQR†)	96.0 (37.0-189)	51.0 (22.0-123)	79.0 (35.0-195)	56.0 (20.0-125)	75.0 (37.0-159)	71.0 (24.0-149)

* RTW= return to work, † IQR= interquartile range

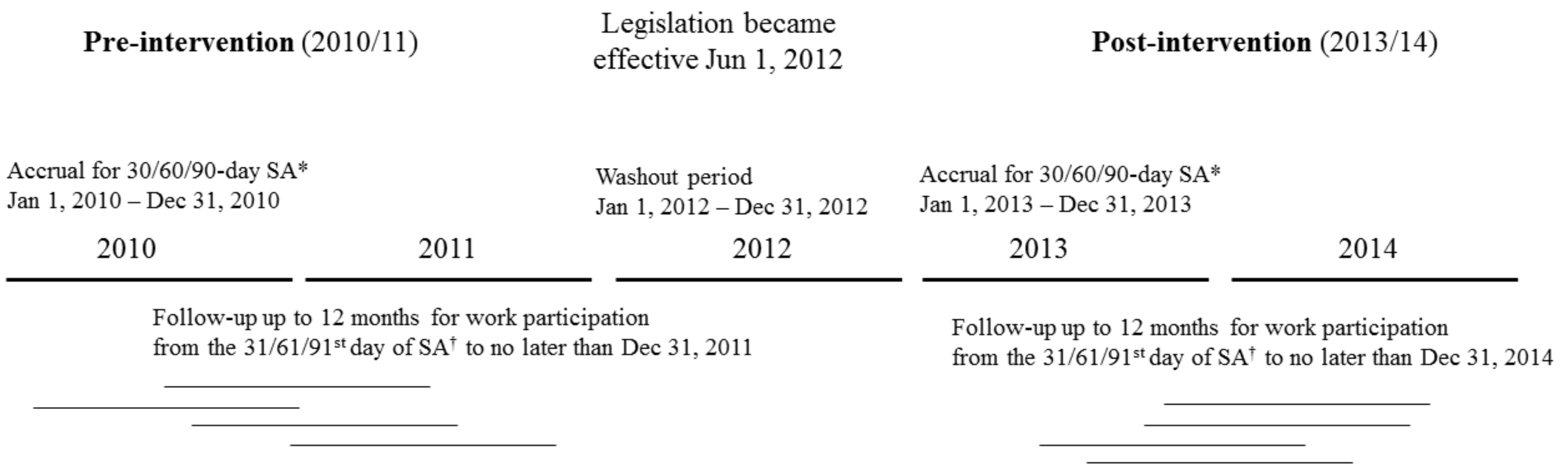

Supplemental Figure 1. Selection of the sickness absence accrual and follow-up periods for the pre- and post-intervention period. The horizontal lines at the bottom indicate follow-up periods for hypothetical individuals.

*corresponding to 30, 81, and 116 calendar days
†corresponding to 31st, 82nd, and 117th calendar day
